# Implementation factors for patient navigation program success: a qualitative study

**DOI:** 10.1186/s43058-021-00248-0

**Published:** 2021-12-20

**Authors:** Mandi L. Pratt-Chapman, Rachel Silber, Jeffrey Tang, Phuong Thao D. Le

**Affiliations:** 1 GW Cancer Center, School of Medicine and Health Sciences, The George Washington University, Washington, DC USA; 2 Graduate School of Arts and Sciences, New York University, New York, USA; 3 School of Global Public Health, New York University, New York, NY USA

**Keywords:** Patient Navigation, Implementation, Sustainability, EPIS Framework

## Abstract

**Background:**

Patient navigation (PN) is an evidence-based practice that involves assessing and addressing individual barriers to care for patients. While PN has shown effectiveness in numerous studies, designing successful, sustainable PN programs has remained challenging for many healthcare organizations. The purpose of the present study was to examine implementation factors for successful PN programs to optimize the sustainability of PN services across cancer care settings in the USA.

**Methods:**

Data were collected via semi-structured interviews with PN stakeholders (*n*=17) from diverse cancer care settings. Thematic content analysis was conducted by deductively coding major themes based on constructs from the Exploration-Preparation-Implementation-Sustainability framework and by inductively coding emergent themes.

**Results:**

Facilitators in the outer context included payer guidelines, accreditation requirements, community partnerships, and demonstrated need and demand for services. Inner context factors such as alignment with organizational and leadership priorities, appropriate staff support and workloads, and relative advantage were important to program success. Innovation characteristics such as the presence of innovation champions, clear role and scope of practice, clear protocols, strong communication channels, and innovation fit were facilitators of program success. Community-Academic partnerships and funding stability also emerged as facilitators for program sustainability.

**Conclusion:**

Our qualitative analysis from a diverse sample of PN stakeholders and programs across the USA supports intentional use of implementation theory to design PN programs to optimize implementation success.

Contributions to the literature

- Patient navigation (PN) is an evidence-based practice that is recommended for cancer care settings; however, little is known about implementation factors that support the success and potential sustainability of PN programs
- Interviews with diverse PN stakeholders suggest that a demonstrated need for PN services, external incentives, external partnerships, internal leadership support, clear role delineation, protocols that fit within the organizational culture and workflow, training supports, and the ability to demonstrate value of services are key factors in PN success and potential sustainability
- Results of this study support intentional use of implementation theory to plan, implement, and evaluate PN programs in order to optimize impact and support the sustainability of services

## Background

Patient navigation (PN) is an evidence-based practice to reduce disparities in cancer outcomes and involves individual assessment and resolution of barriers to care [1]. Cancer PN has been effective in reducing time from screening to definitive diagnosis [2], increasing adherence to recommended treatment [3], and improving patient satisfaction [4, 5]. Research suggests that PN is most effective for patients with clear risk factors associated with delays to care, such as comorbid conditions, low socioeconomic status, and low educational attainment [6]. PN has also been shown to be cost-effective, particularly for colorectal cancer screening programs (i.e., finding and removing pre-cancerous polyps early avoids expensive treatment costs) and among geriatric patients with multiple comorbidities [7, 8]. While there has been a published business case for PN [9] with clear case examples for return on investment, sustainment of PN programs remains challenging for many institutions as it is typically a non-revenue-generating service and thus vulnerable to budget cuts when finances are constrained—such as during the recent COVID-19 pandemic when patient volumes plummeted and resources were redirected to address the virus.

While research on PN effectiveness has grown over the last several decades, few studies have designed or evaluated PN using implementation or sustainability frameworks. Implementation science is a scientific approach to translating research evidence into practice. Two extant studies have explicitly used implementation theory to optimize PN within a particular context: Chicago’s Chinatown study [10] and Boston’s Translating Research Into Practice (TRIP) study [11]. The Chinatown study used the Consolidated Framework for Implementation Research [12] while TRIP referenced implementation principles, but did not cite a particular model. Both studies were collaboratively designed with a community-based coalition to tailor the PN intervention to the specific populations of focus. The Chinatown program emphasized internal leadership, training supports, and strong communication channels. Another study by the Penn Medicine Breast Health Initiative used a quality improvement model to optimize PN and found that securing funding for the program, training navigators and role clarity, community partnerships, culturally-tailored messaging, and evaluation were critical to optimize its program results [13].

The purpose of this study was to examine implementation factors that are related to successful implementation, maintenance, and sustainment of PN programs across diverse cancer care settings. The study focuses primarily on success factors for program implementation and potential sustainability.

## Methods

### Conceptual framework and epistemology

Drawing from a pragmatic epistemology, we conducted a qualitative study using semi-structured video interviews, guided by the Practical Robust Implementation and Sustainability Model (PRISM) [14]. PRISM is one of few implementation science theoretical models that includes sustainability. The model considers how intervention design, external environment, recipients, and infrastructure affect program adoption, implementation, and maintenance. After examining the data, we supplemented PRISM with the Exploration-Preparation-Implementation-Sustainability (EPIS) framework [15], which includes similar constructs, but also includes phases of implementation from preparation through sustainment.

### Researcher characteristics and reflexivity

MPC and PTL are qualitative experts and RS and JT are junior researchers. MPC has a demonstrated track record of published PN workforce capacity research. None of the authors have any direct relationship with research participants.

### Setting and subjects

Patient navigators, PN supervisors, and administrators from cancer centers were purposively recruited based on (1) participating in the Centers for Medicare and Medicaid Services (CMS) Oncology Care Model (OCM) or (2) published PN research. Our goal was to recruit seven institutions with three stakeholders from each institution.

#### Sampling strategy

An attempt was made to recruit one patient navigator, one supervisor, and one administrator from each site to provide varied perspectives on facilitators and barriers that affect program success and sustainability. Up to three recruitment phone calls or emails were sent to each potential participant by a research assistant using a standardized script approved by the PI.

#### Consent

Participants were sent an information sheet about the study, which was also reviewed verbally prior to each interview. Each participant was asked to verbally consent and to indicate permission to record the interview.

### Ethical review

This study was deemed exempt by the George Washington University IRB (#180907).

### Data collection

Semi-structured interviews of approximately 60 minutes each were conducted by a trained research assistant between March and July of 2019; digitally recorded via WebEx; and transcribed through Rev.com (San Francisco, CA). An interview guide consisting of ten questions asked about navigator role, reason for program initiation, services provided, organizational fit, engagement of key decision-makers, level of integration into organizational workflow, staffing, program infrastructure, sharing of evaluation data, and future directions. Questions were intended to mine information about the intervention, implementation and sustainability structure, and organizational characteristics based on PRISM [15].

Interviews were conducted until data saturation was reached. Identifiers were removed from transcripts prior to coding. No incentives were provided.

### Data analysis

RS and JT conducted line-by-line coding using Dedoose software (dedoose.com) with oversight from PTL and MPC. Data were first deductively coded to capture examples of EPIS constructs [16], and then emergent themes were inductively coded. The study team met weekly over a 3-month period to develop consensus in coding excerpts and refine the codebook. RS and JT reviewed 10% of transcripts and agreed on codes. Subsequently, RS and JT conducted primary coding and drafted memos of key themes from half of the remaining transcripts, respectively. All authors reviewed coding for final agreement and chose exemplar quotations.

## Results

### Sample characteristics

Of 21 participants invited to participate, 81% (*n*=17) consented to interview. For one program, a supervisor had recently left, so a physician was interviewed instead. Participants included patient navigators (*n*=7), navigation supervisors (*n*=4), administrators (*n*=5), and physicians (*n*=2) from seven cancer centers of various sizes across the USA. Regionally, one institution was located in the Northeast, two in the Mid-Atlantic, two in the South, and two in the Midwest. While all centers were located in cities, four centers served larger rural areas while three served primarily urban areas. Regional representation is presented by location of individual interviewees rather than institution or state (Table 1). We successfully recruited three stakeholders each in four institutions, two stakeholders each in two institutions, and one stakeholder from one institution.

**Table 1:** Sample characteristics (*n*=17)

Participant characteristics	*N* (%)
Sex	
Female	16 (94.1%)
Male	1 (5.9%)
Role^a^	
Administrator	5 (29.4%)
Navigation supervisor	4 (23.5%)
Patient navigator	7 (41.2%)
Physician	2 (11.8%)
Region	
Northeast	2 (11.8%)
MidAtlantic	5 (29.4%)
South	4 (23.5%)
Midwest	6 (35.3%)
Catchment	
Rural	9 (52.9%)
Urban	8 (47.1%)

^a^Not mutually exclusive

### Themes

The EPIS framework includes outer and inner context factors, innovation factors, and bridging factors that influence the success of innovations. Themes relevant to EPIS constructs are described below with illustrative quotations for themes provided (Table 2).

**Table 2:** Thematic EPIS constructs

EPIS construct	Example quotation
**Outer context**	
Funding, guidelines, and service environment: OCM bundled payments and CoC accreditation requirements	[O]ur participation in our oncology care model and the fact that they require navigation, it has definitely given both financial support and cultural support to continue to grow our navigation program, which has expanded substantially during the past few years. -Navigation Supervisor “[W]e have to present for the American College of Surgeons and that’s the standard [for] PN… [S]ince we already had this in place it’s been a benefit to the hospital.” -Navigation Supervisor
External networks: community partnerships and philanthropy	As a community, we work with the city, we work with the councilmen, we work with the pastors, the barbershops, the beauty shops. The navigators get in there and as we identify patients, they’re the ones that bring them in… You can look at the data: patients do not show up unless they’re navigated through the system. -Administrator Because we’ve been able to show how we’ve helped patients, my chairman...has created a push towards philanthropic dollars to help support some of my activities. -Administrator
Patient characteristics and advocacy: demonstrated need for services	[B]ased on previous research…looking at minority, underserved, English as a second language ’cause those [are] shown to be the people who benefit most from navigation. Yes… we’ve seen a decrease in time to diagnosis, a decrease in time to treatment within the breast center…[W] e are looking at screening adherence and then again time to diagnosis or whether or definitive resolution if it’s not cancer. -Navigation Supervisor They want to be able to provide a cost of the patient’s estimated services…to prevent them from having kind of a shell shock of getting a large bill on the back end…[I]t all ties into just the patient need. I think that that’s a part of the overall care of a patient. It’s not just getting well physically, but just mentally knowing that the bills are being taken care of and that someone is on their side to help maneuver through the billing aspect of it all and insurance aspect of it all. -Patient Navigator [O]ur patients were showing up…with late stage disease…[They] had a lack of education and fear regarding the health systems and trying to get their cancer screenings. So we created our first Navigation Program to help the community improve cancer outcomes and late stage disease presentation. -Administrator
**Inner context**	
Alignment with organizational priorities: strategic priorities and leadership support	[The program] is embedded in our cancer service line strategic initiative to expand and enhance our care coordination services. So we are actually looking to expand and enhance the nurse [and] lay navigation program. -Administrator From the very beginning it was high, high level, highest level leadership that was saying, “This is what’s going to happen, and this is how we’re going to do it”, and so our reporting structure is at the highest levels. -Navigation Supervisor
Organizational support: Training	[W]e can’t just do PowerPoints and recorded classes, because it’s hard for them to get everything they need from that…[O]ur onboarding program…is a combination of… class work that they’re doing with us, online courses, [and] on the job training. -Administrator
Appropriate staffing levels	We are a team of seven…The way that everything is split and divided, I think we have pretty good coverage -Patient Navigator
Relative advantage	It’s helpful in showing the positive impact that we have and just the volume of our work. [Leadership] can actually see, basically, how many patients we’ve interacted with, individual patients that we’ve interacted with over the year...it helps justify the need for [additional] navigator[s]. -Administrator [W]e looked at patients who were navigated versus patients who weren’t navigated, in terms of their retention within the health system, the percentage of patients who went to and received chemotherapy, those who received radiation… [T]his was actually the most profitable program we’ve ever had within the health system. -Navigation Supervisor
**Innovation factors**	
Innovation developers	I hire the navigators, supervise the navigators on a day-to-day basis….Most all of our navigators now are currently grant funded so a lot of the grants are written by me. -Navigation Supervisor
Navigator role and scope of practice	We are all lay navigation, but we partner with nurse navigation programs in the clinic… [W]e’re…at about 250 patients per navigator…for the lay navigators, the clinical navigators [have] a much lower…number. We take first line for them, so they’re doing clinical escalations. -Administrator
Clear protocols	[I]f one of our patients goes to the emergency room, we get a notification...[T]he day after they get back from the emergency room, we’re going to call and find out: What did you go to an emergency room for? Did you call the clinic?…Do you have a follow up appointment scheduled with your doctor? Did you build your scripts? Did they take care of everything? So we don’t want that emergency room visit to result in a hospitalization or another ER visit. -Administrator
Strong communication channels	[W]e have coordinated care between different specialties, and that’s when the navigators come in and talk to the patients about the complexity of care and the need to be seen by multiple providers, and helping us coordinate the tests. So, they have access to the tumor boards. -Medical Oncologist
Innovation fit	[W]hen we start a project or hire a navigator working with the clinic...[W]e meet with the managers and sort of see how is the best way for the navigator to fit in. We make them a part of the team. They meet with social work. And we make it very clear that we have lay navigators. They practice within their scope. They’re not doing any psychosocial sort of thing, but are there to sort of support the social workers. -Navigation Supervisor
**Sustainability**	
Community-academic partnerships	[O]ur researchers are able to say, “Okay, I know that she’s tracking this in head, and neck. She has some information that I would like to look at, and I think it can fund a proposal for a grant.” -Navigation Supervisor
Funding stability	[W]hen somebody says the service is amazing and the doctors want it but the free money runs out and nobody’s running around to try to get more money, that doesn’t make it feel like navigation is an important asset to the organization as a whole. -Patient Navigator

### Outer context

Outer context factors affected the role and focus of the patient navigator as well as program sustainability and growth in our study. For example, service environment and need for services as demonstrated through patient characteristics or community assessments were factors that shaped the role and focus of PN tasks. External networks and accreditation policies were drivers of growth and sustainability.

Overall, financial support of the OCM, American College of Surgeons’ Commission on Cancer (CoC) accreditation requirements, and internal funding for PN programs were facilitators for program success and sustainability. For programs that were participating in the OCM, payer requirements directly influenced the service environment and incentivized clear navigation protocols for assessment and monitoring. Overall, OCM requirements strongly influenced the stability, focus, and processes for participating programs, since payment relied on meeting quality indicators. External funding through grants, however, shifted the focus away from patient care to reporting and meeting the requirements of the funding source. For example, one program initially was funded by CMS to reduce costs among geriatric patients, but when funding ended, the program shifted to meet OCM requirements in order to sustain PN services. Internally funded navigators (programs funded through operational funds) were more likely to be engaged in ongoing quality and process improvements. In addition, internally funded programs that were centrally structured reported greater efficiency and autonomy to focus on navigation. While not a funder, the CoC PN standard for its accredited programs also drove program initiation and sustainment.

Within EPIS, external partnerships are both outer context and bridging factors. Our data indicated that PN partnerships with community organizations yielded improved adherence to care. Philanthropy was also a noted facilitator to meet patient needs by providing free screenings and transportation, and supporting patients facing significant financial toxicity due to their cancer treatment—such as mortgage payments, utility bills, and car payments.

EPIS constructs of patient advocacy and patient characteristics align with our findings for demand or demonstrated need for services. Determination of need for services varied and included internal assessment, community assessment, desire to increase diversity in clinical trials, and accreditation requirements. Reasons for program initiation varied: For example, a navigation supervisor described how their program was initiated as a result of prior research that explored the challenges of individuals whose primary language was not English. A patient navigator indicated the need for navigation to help patients understand insurance and financial impacts of their cancer treatment: In some cases, patients were declining treatment due to cost.

Some programs were initiated specifically to address the complexities of varying forms or stages of cancer. An administrator at a large center reported that their program started in order to reduce late-stage diagnoses. In each scenario, program initiation was driven by an identified barrier to cancer care adherence.

### Inner context

Sustainability factors related to inner context include organizational characteristics, culture, and internal leadership; organizational staffing and processes; individual characteristics; and the ability to show relative advantage of the navigation program through evaluation.

A key facilitator for program success was the importance of aligning program goals with organizational strategic initiatives, leadership priorities, and organizational culture. Internal leadership was noted as a facilitator for program initiation and growth. For example, an administrator reported that a new CEO at the institution encouraged employees to engage in community outreach, and thus, the program aligned with the CEO’s strategic priority. Alignment with disease teams and goals was also a facilitator for success. Program alignment led to greater satisfaction within the navigation team and a sense of being valued.

Strong programs established on-the-job and online training on-boarding processes and appropriate staffing levels. Patient navigators at institutions that did not invest in their professional development suffered. Appropriate levels of staffing with reasonable workloads also surfaced as important. In contrast, staffing inadequacies led to challenges in fitting with clinic workflow. Likewise, lack of staffing slowed efficiency: One navigation supervisor noted challenges with obtaining records for complex cases due to understaffing which led to delays in care.

Data collection among participating sites was essential to show the relative advantage of PN services. Evaluation data were collected in a variety of ways, including Excel, REDCap, Electronic Health Records (EHRs), and a customized app. Data were used to show program impact internally to leadership and supported program growth in alignment with patient needs. Data were used to show productivity, resolution of patient barriers to care, and patient retention that led to indirect revenue. A program with robust evaluation was able to show value in terms of patient retention and services billed. In contrast, a program that did not prioritize data collection reported a lack of institutional support or prioritization of navigation services. This same navigator noted a lack of internal support for program sustainment.

### Innovation factors

Prominent innovation factors for sustainability included innovation developers, innovation characteristics, and innovation fit. Innovation developers varied by program, but typically fell to the navigation supervisor who distinguished roles within patient services and championed navigation services. The importance of a dedicated role for program development was a common theme. Programs varied in emphasis on nurse versus non-clinical PN, but clear distinctions between these roles was a success factor. Program stakeholders reported navigation tasks including arranging transportation, facilitating screenings, resolving insurance challenges, finding and connecting patients to community resources, resolving challenges with food insecurity or utility payments, arranging for co-pay or free drug assistance, assessing eligibility for financial assistance, arranging genetic testing, arranging interpretation services, scheduling future appointments, preparing patients for visits with a provider, educating patients about what treatment involves and what services are available, assessing patient self-efficacy to take the steps needed to adhere to treatment, assessing distress, helping patients organize information regarding follow up tests and how the system works, and following up with patients by phone or in person to remind patients of appointments and services available. Additional tasks included obtaining referrals for services, identifying past medical history and services and procedures a patient has received, charting in the medical record, and contacting primary care providers to report back on procedures a patient received or is recommended to receive.

Facilitators for program success included team coordination; collaboration within an appropriate scope of practice; clear protocols; strong communication channels; and innovation fit to context. A Medical Oncologist reported that lack of clear role delineation resulted in role confusion and staff not operating at the top of their license. Clear protocols and scripts aided navigation efficiency and optimized patient care coordination to avoid expensive emergency room visits. Strong communication channels also emerged as an important characteristic for program success. Patient navigator participation in multi-disciplinary team meetings helped foster team communication. Inclusion of patient navigators in team meetings and tumor boards provided them with information to better coordinate care for patients. In contrast, barriers to communication resulted in less efficient care coordination. Thoughtful integration into existing workflows was also a factor for success.

### Sustainability

Also a bridging factor, internal collaboration with researchers was noted as a strategy to optimize external funding support, and thus the sustainability of programs. Community-academic partnerships were not prevalent, but some programs voiced aspirations to integrate navigation into more community outreach and research in the future. The benefit of community-academic partnerships, when present, included robust evaluation showing the value of PN—indirectly supporting the sustainability of the intervention.

Funding stability emerged as the most important indicator for sustainability. Diverse funding streams were reported, including grants, operational budgets, and OCM financing.

Navigators with internal funding expressed perceived job security. Conversely, when PN was contingent on grant funding, sustainment was jeopardized and navigators felt devalued.

## Discussion

A critical challenge to PN sustainability is lack of financing for services. Historically, PN programs have been supported by grants with a limited duration and short-term outcomes. For example, the National Institutes of Health (NIH), the Centers for Disease Control and Prevention (CDC), the Centers for Medicare and Medicaid Services (CMS), and the Health Resources and Services Administration (HRSA) have funded numerous research studies to support demonstration projects and evaluate the efficacy of PN [17]. The American Cancer Society (ACS), the Avon Safety Net Foundation, and Susan G. Komen have supported PN in practice through grant funding. However, grants from foundations have declined in recent years [18]. Thus reliance on grants is not a long-term sustainability strategy.

Integration of PN into standard of care is required for ongoing sustainment. Value-based payment models are promising options for PN sustainability. For example, the OCM requires institutions to include PN as one of several essential services required for payment [19, 20]. A $160 up-front per patient per episode payment is provided to offset the costs of these services [19]. In other disease areas, limited PN has been paid for under Medicare or Medicaid fee-for-service (FFS) codes [18]. These programs typically focus on patient education regarding diagnosis or self-management of a disease, limiting the flexibility of PN services as well as the patient populations eligible to receive services. The inflexibility of FFS payments, however, makes value-based options more attractive for oncology PN programs. Nevertheless, an FFS option for cancer screening might be quite helpful to improve adherence to guideline-driven screening.

Regardless of the financial source of support, PN programs will only be sustained if leadership within cancer care organizations value PN services. With leadership support, PN can not only be sustained, but grow. For example, Sarah Cannon, the Cancer Institute of HCA Healthcare, has demonstrated increases in patient volumes, less staff turnover, and greater patient satisfaction—leading to increasing support for an ever-growing PN program of services [9]. Their progressive leadership is an exemplar in using evaluation data to support growth.

Finally, the variability of PN programs can make it challenging to draw generalizable conclusions for best practices—in turn, making it difficult to know what exactly should be sustained. Some cancer centers employ nurse navigators and others employ patient navigators without a clinical license. The scope of practice of each of these types of navigators varies. In addition, patient needs might demand different services from navigators. Where PN may be driven by the linguistic needs of some patient populations across the continuum of care, PN may be focused on reducing late-stage diagnoses in screening settings. Some centers employ a team-based approach, triaging patients to the appropriately skilled navigator based on the type of patient need. While common tasks of PNs include providing education to patients, identifying and addressing barriers to care, assisting with scheduling, attending appointments with patients, and providing referrals for services, patient needs across settings vary widely [21].

Our study contributes to the PN literature in several ways. We fill a gap in the literature by using implementation theory to identify factors across settings that aid in implementation success and sustainability. Specifically, this research is the first study to our knowledge to use the EPIS framework to describe PN program success and sustainability. In our study, a number of clear factors to program success and sustainability emerged that align with three of four phases of the EPIS framework. For the Preparation phase of EPIS, demonstrated need for services based on individuals served by the system and leadership support were critical factors. For the Implementation phase, alignment with organizational culture and strategic priorities, provision of training and staff supports, clear role delineation among team members, dedicated program leaders, clear protocols and scripts for navigators, and strong communication channels proved helpful for PN programs. For example, innovation fit was optimized in one center by clearly delineating between PN and social work roles (see Table 2, Innovation fit). Bridging factors such as relationships with community-based organizations further supported successful implementation—for example, reliance on established faith-based networks to drive patients to cancer screening. This finding aligns with a recent review that found partnerships to be essential in the development of community health worker and PN programs [21]. Sustainment was facilitated by ability to show relative advantage through evaluation as well as external incentives such as payer or accreditation requirements. Financing streams varied, but funding stability was the clearest facilitator for program sustainment.

Our study found that noted barriers to PN program success included inconsistent funding, lack of a program champion with authority, ineffective training or lack of professional development for staff, and inadequate staffing and workloads. For example, programs that depended on grant funding were vulnerable to reduced staffing over time. Many grant-funded programs had an abundance of patients and a dearth of navigators. Navigators expressed feeling overwhelmed trying to meet funder requirements. For programs where grant funding ultimately was exhausted, some programs lost staff, ultimately reducing patient support and care coordination efficiency.

It is worth noting explicitly that a fundamental assumption of our study is that strong, successful programs will be more sustainable. In the primary care literature, a recent scoping review of PN programs identified eleven core elements of sustainable navigation programs, all of which align with our qualitative findings regarding successful programs [22]. These include attention to patient characteristics, training of navigators, role clarity, clear operational processes, adequate staffing and resources, strong inter-organizational networks, community partnerships, strong communication channels, demonstrated need for the program (i.e., program uptake and buy in by end users of the program), valuing of navigators, and evaluation (i.e., showing the value of the program). Our study provides additional support for the findings of this scoping review and suggests that these sustainability factors are transferable to PN programs across various clinical areas.

### Strengths

As noted above, the use of the EPIS framework specifically, and implementation science theory generally, are strengths of our study. Another strength is the examination of constructs from multiple perspectives—not just that of navigators, but also program supervisors and administrators.

### Limitations

This study was limited by a small sample of navigation programs that may have represented more robust programs than are typical due to participation in the OCM and extant publications. Our sampling approach did not allow for comparisons among a similar number of successful and unsuccessful programs. Recruitment of interviewees was also challenging given the lack of incentives.

### Future research directions

Future research is warranted to identify specific and parsimonious evaluation metrics critical to PN program sustainability. Additional work to tie evaluation to payer metrics will be critical for sustainability. Additional research is also needed to identify optimal care coordination strategies for navigators in collaboration with clinicians and community-based organizations, including communication and documentation through health information exchanges. Furthermore, research on whether PN increases adherence to treatment by moderating patient financial toxicity is warranted and would be novel. While our study attempted to identify important implementation constructs, more in-depth observational research may be warranted to provide even richer data regarding important variations among PN program settings. Longitudinal observational studies will be important to confirm the preliminary insights on sustainment we found in our study.

## Conclusion

PN can be a cost-effective way to address barriers in access to cancer care. We identified several major facilitators for PN sustainability: aligned external incentives, strong leadership, clear roles and protocols, and effective partnerships tailored to patient needs. Additional facilitators were demonstrating a need for the program, clear communication channels, training supports, and funding stability. Without these factors, patient navigators may feel unsupported and undervalued and programs may experience challenges to sustainment.

## Data Availability

The datasets used and/or analyzed during the current study are available from the corresponding author on reasonable request.

## Abbreviations

CoC: Commission on Cancer
CMS: Centers for Medicare and Medicaid Services
EPIS: Exploration Preparation Implementation Sustainment
OCM: Oncology Care Model
PRISM: Practical, Robust Implementation and Sustainability Model
